# Chasing Ecological Interactions

**DOI:** 10.1371/journal.pbio.1002559

**Published:** 2016-09-15

**Authors:** Pedro Jordano

**Affiliations:** Integrative Ecology Group, Estación Biológica de Doñana, EBD-CSIC, Av. Americo Vespucio s/n, s/n, E-41092 Sevilla, Spain

## Abstract

Basic research on biodiversity has concentrated on individual species—naming new species, studying distribution patterns, and analyzing their evolutionary relationships. Yet biodiversity is more than a collection of individual species; it is the combination of biological entities and processes that support life on Earth. To understand biodiversity we must catalog it, but we must also assess the ways species interact with other species to provide functional support for the Tree of Life. Ecological interactions may be lost well before the species involved in those interactions go extinct; their ecological functions disappear even though they remain. Here, I address the challenges in studying the functional aspects of species interactions and how basic research is helping us address the fast-paced extinction of species due to human activities.

## Abstract 2

Pedro Jordano assesses the challenges of studying the functional aspects of species interactions and how basic research is helping us to address the current fast-paced extinction of species due to human activities.

I am tempted to give one more instance showing how plants and animals, most remote in the scale of nature, are bound together by a web of complex relations.

(Darwin, Charles. 1860. On the origin of species by means of natural selection. Chapter 3, p. 75).

There is a much more insidious kind of extinction: the extinction of ecological interactions.

(Janzen, D.H. 1974. The deflowering of Central America. *Natural History*. 83:48–53).

Suppose you want to build the LEGO Triceratops Trapper model. It has 256 pieces, corresponding to 74 distinct parts. It’s a relatively simple model, yet impossible to build by assembling these 256 pieces at random. To build the fully functional Triceratops Trapper we need to know more than the inventory of its parts: we also need to know how the different pieces fit together. We’ll have a functional Triceratops Trapper only if we assemble the model connecting its component pieces the right way. Like the pieces of our Triceratops Trapper, species in ecosystems are not connected (linked) by random interactions. They fit together to form functional units that share some basic properties independent of the type of ecosystem and even of the type of ecological interaction. This Web of Life [1] shapes the wireframe that supports biodiversity, and ecological interactions among the species that make up this web provide essential services and functions that support its persistence. Interactions might be lost (go extinct) even well before the species. For instance, the “empty forest syndrome” [2] describes situations in which animals and plants may persist in disturbed areas (e.g., a tropical forest fragment) yet in such reduced abundance that their functional ecological role is lost. Interaction extinction may result in the loss of important ecological functions, such as pollination and seed dispersal, that are crucial for forest regeneration and ecosystem persistence. These losses of interactions cause unprecedented changes in cascade in natural communities (i.e., like trophic cascades), implying losses of ecological functions. For example, just imagine the myriad consequences of extinctions of pollinators and frugivore seed dispersers for ecosystems like tropical rainforests, where more than 90% of the woody plant species depend on frugivores to support their life cycles. The disappearance of frugivores could unleash a cascade of effects with unprecedented changes in the structure and function of an ecosystem.

No single species on Earth lives without interacting with other species. These interactions are the backbone of biodiversity and create the architectural foundation of ecosystems. Exploring and inventorying biodiversity represent a fundamental challenge for basic research in ecology and conservation biology. This basic knowledge is urgently required to properly diagnose the status of biodiversity conservation and develop early warning signals for the potential disappearance of interactions and collapse of communities.

## Diversity of Species and Their Interactions

**Fig 1:**
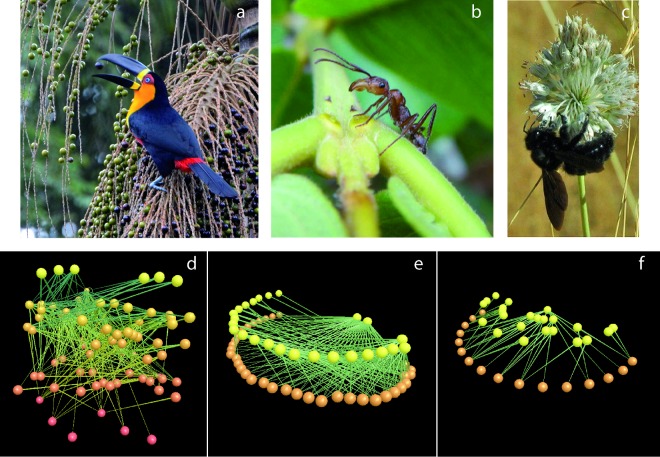
The structure of ecological interactions. Top: examples of ecological interactions between plants and animals. (A) Ariel toucan (*Ramphastos ariel*) (Ramphastidae) feeding on palmito juçara fruits (*Euterpe edulis*) (Arecaceae) in the Brazilian Atlantic rainforest. (B) *Ectatomma tuberculatum* over extra-floral nectaries at the base of a leaf of *Qualea multiflora* (Vochysiaceae) in the cerrado vegetation (Brazil). (C) A carpenter bee (*Xylocopa violacea*) (Apidae) visiting an *Allium ampeloprasum* (Liliaceae) (wild garlic) inflorescence in Southeast Spain. Bottom: different visualizations of the complexity of interaction networks among species (colored spheres) illustrated by their actual links (light green lines). (D) Food webs typically describe all the interactions occurring in a given ecosystem with multiple trophic levels. (E) Most plant–animal interactions can be displayed as bipartite graphs describing the pairwise pattern of mutual interdependencies among two distinct sets of animals (orange nodes) and plants (yellow nodes). (F) Interactions among species with a higher degree of intimacy, such as ant–plants, show a distinct pattern of structure, often with multiple distinct groups (modules) of closely intimate associations. Image credits: (A) José Augusto Balieiro, with permission; (B) Denise Lange and Kleber del-Claro, with permission; (C) Pedro Jordano. Panels D, E, and F were produced with FoodWeb3D, written by R.J. Williams, and provided by the Pacific Ecoinformatics and Computational Ecology Lab (www.foodwebs.org).

The Web of Life results from the assembly of species that interact with each other in a variety of ways, forming complex interaction networks (Fig 1). A myriad of interaction modes exists in nature, reflecting the complexity of natural histories of partner species. Interactions take the form of predation, competition, commensalism, amensalism, mutualism, symbiosis, and parasitism and, in all cases, involve reciprocal effects for the interacting species. Recent basic research on the topology and structure of these networks has revealed universal patterns that ultimately affect their stability and resilience. Yet we are far from fully documenting all the types of interaction modes that exist, even in simple ecosystems.

Just as we sample individuals of free-living species to estimate the diversity in a particular area or ecosystem, we can sample interactions. In this way, we can assess the full complexity of ecosystem structure. Yet the exercise is not trivial. We are far from fully understanding the minimum set of functional links that are needed to support and restore damaged ecosystems. We don’t even have robust estimates of the total number of species living on Earth. Assessing the diversity of their interactions is a far more daunting task.

Life on Earth is supported by zillions of interactions among species. Understanding these complex systems demands that a large fraction of these interactions be experimentally or computationally probed. This is very difficult, as rapid and effective actions or conservation and restoration of human-disturbed ecosystems urgently require the identification of the minimum amount of complexity that has to be restored to facilitate an ecosystem’s persistence.

Considering all the distinct ways in which such highly complex, interactive systems can be decomposed into parts cannot be done on a statistical basis (as, for example, with an ideal gas), because each interaction is particular; genomes, proteins, cells, and species interact in specific ways. The number of ways a complex system can be partitioned is known as Bell’s number (Bn) [3]. For three elements, there are B_3_ = 5 such partitions; three species may not interact at all, or any two of them can interact, or all three, for a total of five possibilities. Understanding the system requires measuring the probability and magnitude of each of these five. This is out of the feasibility limits for any study [3], given that Bell’s number scales supra-exponentially with the number of components. In real plant–animal webs, the number of actual pairwise interactions among species in local assemblages scales with species richness (Fig 1). These real ecological systems would be within the range of *n* = 10^3^*−*10^5^ or even *n* = 10^4^*−*10^6.5^ components, depending on spatial scale when we move from local to regional and up to continental spatial scales [4]. To fully quantify the size of these interactomes, we thus need to focus on what we know about the macroscopic properties of complex ecological interaction networks [1].

## Basic Conservation Science in the Anthropocene: Challenges

Most discussions about the effects of the biodiversity crisis have largely focused on the loss of species, ignoring the extinction of ecological interactions. In our lifetimes, a myriad of vertebrate species has experienced population declines and eventual extinctions matching human expansion, i.e., the “Anthropocene defaunation” [5]. Large-bodied vertebrates have been especially hard hit, and, as a result, many disturbed ecosystems currently host only small- to medium-bodied species, or population sizes so reduced that the species are no longer functional in their ecosystem [2]. If the remnant, extant species fail to provide pivotal services formerly assisted by vanishing large vertebrates, human-driven defaunation may trigger negative cascading effects on ecosystem dynamics. This downgrading process is expected to result, for example, in collapsed mutualisms of pollination and seed dispersal for plants depending on them for regeneration.

Early signals of the collapse of mutualistic interactions may trigger severe population declines, which lag behind the loss of seed dispersers or pollinators. Consider, for example, that deforestation, logging, fragmentation, and climate change are already having significant impacts on tropical carbon stocks. Further reductions in carbon storage could follow the disruption of mutualisms resulting from the loss of animal pollinators and frugivores acting as seed dispersers for diverse forest plants, because forest regeneration may collapse. Loss of key ecological interactions may precede the local extinction of partner species that depend on the key ecological services provided. Without basic ecological and biodiversity research, we’d have no hope of forecasting the consequences and rebuilding functional ecosystems.

## Abbreviations

Bn: Bell’s number

## References

[1] Thompson JN. The coevolving web of life. The American Naturalist 2009; 173:125–140. 10.1086/595752 19119876

[2] Redford KH. The empty forest. Bioscience. 1992; 42: 412–422.

[3] Koch C. Modular biological complexity. Science. 2012; 337: 531–532. 10.1126/science.1218616 22859475

[4] Lima-Mendez G, Faust K, Henry N, Decelle J, Colin S, Carcillo F, et al Ocean plankton. Determinants of community structure in the global plankton interactome. Science. 2015; 348: 1262073–1262073. 10.1126/science.1262073 25999517

[5] Dirzo R, Young HS, Galetti M, Ceballos G, Isaac NJB, Collen B. Defaunation in the Anthropocene. Science. 2014; 345: 401–406. 10.1126/science.1251817 25061202

